# Epidemiology and risk factors of multidrug-resistant organisms in a Chinese tertiary hospital: a 10-year retrospective cohort study across the COVID-19 policy phases, 2013–2023

**DOI:** 10.3389/fcimb.2026.1786780

**Published:** 2026-04-07

**Authors:** Shanshan Yang, Meng Wei, Haixu Chen, Yazhuo Hu, Zheng Luo, Tingjian Cao, Xintong Nie, Ya Qiu

**Affiliations:** 1 Institute of Geriatrics, The 2nd Medical Center, Beijing Key Laboratory of Aging and Geriatrics, National Clinical Research Center for Geriatrics Diseases, Chinese People’s Liberation Army (PLA) General Hospital, Beijing, China; 2 Department of Disease Prevention and Control, The 1st Medical Center, Chinese People’s Liberation Army (PLA) General Hospital, Beijing, China; 3 Department of Pulmonary and Critical Care Medicine, The 2nd Medical Center, Chinese People’s Liberation Army (PLA) General Hospital, Beijing, China

**Keywords:** COVID-19, CRAB, CRE, hospital-acquired infection, multidrug-resistant organisms

## Abstract

**Background:**

Multidrug-resistant organism (MDRO) infections are a major global health threat. The COVID-19 pandemic and related containment policies may have altered hospital infection epidemiology and the distribution of MDROs.

**Methods:**

In this retrospective cohort study, clinical and microbiological data from 2,669 adult inpatients with culture-confirmed hospital-acquired infections between 2013 and 2023 were analyzed. MDROs were defined per CLSI guidelines. The study period was stratified into pre-pandemic, strict containment, and post-adjustment phases. Multivariate logistic regression was used to identified factors associated with specific MDRO infections.

**Results:**

The overall MDRO detection rate increased from 36.9% to 56.4% during the strict containment. CRAB, which had previously been the dominant resistant pathogen, was progressively overtaken by CRE, while *Klebsiella pneumoniae* became the predominant species in 2022 (40.0%).Bloodstream infections increased from a pre-pandemic peak of 24% to 32% during the strict containment phase. Male sex, age≥60 years, and prolonged hospitalization (>14 days) were associated with distinct distribution patterns of CRAB and CRE infection. In multivariable analyses, mechanical ventilation was associated with increased odds of CRAB infection (aOR=2.07, 95% CI: 1.36-3.16), while urinary catheter use was associated with increased odds of CRE infection (aOR=1.49, 95% CI: 1.06-2.09) but lower odds of CRAB infection (aOR=0.64, 95% CI: 0.46-0.89). Endotracheal intubation was associated with increased odds of VRE infection (aOR = 5.84, 95% CI: 1.10-30.9).

**Conclusions:**

Hospital MDRO epidemiology shifted substantially across the COVID-19 policy phases, with CRE emerging as the dominant resistant pathogen. Specific invasive procedures showed pathogen-specific associations, supporting risk stratification and more targeted infection prevention strategies in high-risk hospitalized patients.

## Introduction

Multidrug-resistant organisms (MDROs) are generally defined as microorganisms that exhibit acquired resistance to multiple classes of antimicrobial agents, commonly defined as non-susceptibility to at least one agent in three or more antimicrobial categories (Magiorakos et al., 2012). MDROs represent a clinically important subset of the broader antimicrobial resistance (AMR) crisis and pose a major challenge to global healthcare systems. Recent global estimates indicate that bacterial AMR was directly responsible for approximately 1.27 million deaths and associated with nearly 4.95 million deaths worldwide in 2019 (Antimicrobial Resistance Collaborators, 2022). The burden of antimicrobial resistance is particularly substantial in low- and middle-income countries, including China. A recent national analysis estimated that approximately 160,000 deaths were directly attributable to AMR in China in 2021, with more than 700,000 deaths associated with resistant infections (Xu et al., 2026). Surveillance data have also reported high resistance rates among key hospital pathogens, particularly carbapenem-resistant *Enterobacteriaceae* (CRE) and carbapenem-resistant *Acinetobacter baumannii* (CRAB), which are considered critical priority pathogens due to their limited treatment options and high mortality (Tacconelli et al., 2018). Identifying modifiable risk factors for MDRO acquisition is therefore of major clinical importance. Understanding which patients are at highest risk and which clinical procedures contribute most strongly to transmission can support targeted infection control strategies and antimicrobial stewardship interventions. Such risk stratification may help optimize empirical antimicrobial therapy, guide surveillance practices, and improve resource allocation in healthcare settings.

The COVID-19 pandemic has further complicated this landscape, as public health interventions likely altered MDRO transmission dynamics (Sun and Fisher, 2021; Amodio et al., 2024).While international reports indicate significant shifts in resistance patterns during the pandemic, including increases in pathogens like CRE and CRAB in some settings (Abubakar et al., 2023; Karakosta et al., 2024), the evidence remains inconsistent. Critically, the long-term impact of specific containment policies on the epidemiology and ecological competition of these key MDROs remains poorly understood.

China’s distinct and phased COVID-19 containment strategy provides a critical opportunity to examine how large-scale public health interventions may influence the epidemiology of MDRO infections. The clear policy transition from “Class A management of Category B infectious diseases” to routine “Category B management” (National Health Commission of the People's Republic of China, 2020; National Health Commission of the People's Republic of China, 2022) directly altered healthcare resource allocation, patient case-mix, and clinical workflows, thereby potentially influencing the infection risks of critical pathogens like CRE and CRAB. While national surveillance networks like CHINET provide broad overviews (Wang, 2022) and multi-center studies have detailed clinical risk factors for specific pathogens (Liu et al., 2020), significant gaps remain in the current understanding of MDRO epidemiology in China. Most critically, there is a paucity of long-term, hospital-based longitudinal data that spans distinct phases of public health interventions. Without such data, it is difficult to disentangle the effects of policy changes from natural secular trends in pathogen transmission. A crucial gap remains in systematically identifying which inpatients are at the highest risk for specific MDROs and which routine clinical procedures are the key drivers of infection across different policy phases. Clarifying these questions is fundamental for developing targeted and actionable infection prevention strategies, yet robust, long-term clinical data to guide such interventions are currently lacking.

To address these gaps, we conducted a 10-year retrospective cohort study (2013-2023) spanning the pre-pandemic, strict containment, and post-adjustment phases. The primary outcomes of this study were infections caused by carbapenem-resistant *Enterobacteriaceae* (CRE) and carbapenem-resistant *Acinetobacter baumannii* (CRAB), the two most prevalent MDROs in our setting. We hypothesized that the risk of CRE and CRAB acquisition varied across COVID-19 policy phases and was independently associated with specific invasive procedures, with distinct pathogen-procedure relationships. This extended timeframe allows us to not only capture secular trends but also to assess the sustained impact of pandemic-era policy changes on MDRO epidemiology. By analyzing a decade of surveillance data across distinct policy periods, this research seeks to provide actionable evidence for refining risk stratification and targeting infection prevention measures in clinical practice.

## Methods

### Study design and setting

This single-center, retrospective cohort study was conducted at a tertiary teaching hospital in Beijing. With a total of 3,800 beds, the hospital accommodates patients in approximately 100 general wards (35–40 beds each) and 10 intensive care units (15–20 beds each). The study consecutively enrolled adult inpatients who were admitted through the hospital’s Emergency Department and subsequently developed clinically meaningful bacterial infections during their hospital stay, between January 2013 and December 2023. Of note, starting from 2020, all patients presenting with fever or respiratory symptoms at the ED underwent SARS-CoV-2 nucleic acid testing according to standard protocols, a practice maintained throughout the COVID-19 pandemic period.

### Study period stratification

The study period was divided into three phases according to national COVID−19 prevention and control policies in China. The pre−pandemic phase spanned from January 1, 2013 to January 19, 2020. The strict containment phase extended from January 20, 2020 to January 7, 2023, corresponding to China’s policy of classifying COVID−19 as a Category B infectious disease but implementing prevention and control measures for Category A infectious diseases (“Category B managed with Category A measures”, hereafter Cat−B[A]), according to the official announcement of the National Health Commission issued on January 20, 2020 (National Health Commission of the People's Republic of China, 2020). Under this policy framework, stringent public health interventions were implemented, including mandatory case reporting within two hours, isolation of confirmed cases, quarantine of close contacts, and strengthened infection prevention and control measures in healthcare settings. The post−adjustment phase covered January 8 to December 31, 2023, following the policy adjustment announced by the National Health Commission on December 26, 2022 (National Health Commission of the People's Republic of China, 2022), which changed the management of COVID−19 to Category B infectious disease under Category B management (hereafter Cat−B[B]). This transition marked the relaxation of strict containment measures and a return to routine infectious disease control strategies. These policy transitions were used to stratify the study period because they substantially influenced healthcare delivery, infection control practices, patient admission patterns, and antimicrobial use in hospitals, thereby potentially affecting the epidemiology of multidrug−resistant organisms. The relatively longer pre-pandemic baseline was included to establish stable epidemiological trends prior to the pandemic, thereby allowing clearer interpretation of the changes observed during the shorter pandemic phases.

### Participant selection

The study cohort comprised adult patients (≥18 years) whose hospitalization originated from an Emergency Department admission and who had at least one clinically meaningful positive bacterial culture during their inpatient stay. Exclusion criteria were: (1) incomplete clinical documentation, (2) repeated hospitalizations (only the first admission was retained for analysis to ensure statistical independence), and (3) bacterial isolates judged by clinicians as colonizers rather than pathogens causing infection. For patients with multiple positive cultures within a single admission, the analysis was conducted at the patient level, focusing on the first detected MDRO event to avoid within-patient correlation.

### Data sources and variables

Data were extracted from the hospital’s integrated electronic sources: the Hospital Information System (HIS), the microbiology laboratory repository, and the electronic medical record (EMR) platform. Collected variables included patient demographics, infection site, specimen type, microbial culture and antimicrobial susceptibility results, primary and secondary diagnoses, treatment records, and details of invasive procedures performed during hospitalization. Two investigators independently performed data extraction and validation, with any inconsistencies adjudicated by a senior third researcher.

### Ethical considerations

The study protocol received ethical approval from the Medical Ethics Committee of the Chinese PLA General Hospital (Approval No. S2019-142-02; Date of approval: September 26, 2019). The requirement for individual informed consent was waived due to the retrospective, anonymized nature of the data analysis.

### Microbiological methods

The collection, transportation, and processing of clinical specimens (including blood, sputum, urine, wound secretions, etc.) strictly adhered to national standard clinical laboratory procedures. Bacterial identification was performed using the VITEK 2 Compact automated microbial identification system (BioMérieux), with confirmation by matrix-assisted laser desorption/ionization time-of-flight mass spectrometry (MALDI-TOF MS) when necessary. Antimicrobial susceptibility testing (AST) was conducted using the broth microdilution method. Results were interpreted according to the Clinical and Laboratory Standards Institute (CLSI) guidelines (M100-Ed33, 2023) (Clinical and Laboratory Standards Institute, 2023).

### MDRO definitions and categorization

The MDROs investigated in this study mainly included CRAB, CRE, methicillin-resistant *Staphylococcus aureus* (MRSA), pandrug-resistant *Pseudomonas aeruginosa* (PDR-PA), vancomycin-resistant enterococci (VRE), and pandrug-resistant *A. baumannii* (PDR-AB). Definitions were based on antimicrobial susceptibility testing interpreted per CLSI guidelines (M100-Ed33, 2023). CRE and CRAB were defined as resistance to any carbapenem; MRSA as oxacillin resistance. The pandrug-resistant (PDR) phenotype was defined as non-susceptibility to all routinely tested antimicrobial categories.

### Clinical data collection

Clinical data were systematically collected from the Hospital Information System (HIS) using structured electronic case report forms. Collected variables included demographic information, clinical characteristics, risk factors, and infection-related parameters. To ensure diagnostic accuracy, all suspected infection cases were independently adjudicated by two infection control physicians in accordance with U.S. CDC/NHSN (Horan et al., 2008) surveillance definitions, with discrepancies resolved through consensus or arbitration by a third specialist.

Following data entry, a dedicated researcher performed structured logic checks to ensure data quality. For each patient (retaining only the first hospital admission), we verified that the infection indicators (e.g., pathogen isolation or specific clinical signs) became positive ≥48 hours after admission. This specific time window, consistent with CDC/NHSN criteria (Horan et al., 2008), strictly distinguished hospital-acquired infections from community-acquired infections that may have prompted admission. Furthermore, for patients with multiple positive cultures or distinct MDRO events during the same qualifying admission, only the first detected event was retained to maintain the patient as the unit of analysis. This three-step approach ensures statistical independence (one admission per patient), etiological clarity (infections confirmed during hospital stay), and avoids overrepresentation of recurrent isolates. Additional checks confirmed that ICU stay duration did not exceed the total length of hospitalization.

### Statistical analysis

Multivariate logistic regression was used to identify independent risk factors for specific MDRO infections. Separate models were constructed for each MDRO outcome (CRE, CRAB, MRSA, VRE, PDR-PA, PDR-AB). All analyses were performed at the patient level, with only the first MDRO event per patient included. This approach ensures that patients with multiple isolates were not overrepresented and that the independence assumption underlying logistic regression was satisfied.

Candidate variables for multivariable analysis were selected based on a combination of clinical relevance and statistical significance in univariate analyses (*P* < 0.10). Age and sex were retained regardless of their univariate P values based on established epidemiological importance as essential demographic confounders in MDRO research. The final models included the following variables: age, sex, hypertension, diabetes, renal insufficiency, cerebrovascular disease, cardiovascular disease, gastrointestinal diseases, trauma, tumor, fever, abdominal pain, disorders of consciousness, dyspnea, acute critical syndromes (none, sepsis without shock, septic shock, non-septic shock), admission department, prolonged hospitalization (>14 days), mechanical ventilation, urinary catheterization, central venous catheterization, endotracheal intubation, and chemotherapy.

To assess multicollinearity among clinically related variables, we calculated the variance inflation factor (VIF). All variables had VIF values < 5, indicating no significant multicollinearity. Model fit was evaluated using the Hosmer-Lemeshow goodness-of-fit test, with *P* > 0.05 indicating acceptable calibration. Discriminative ability was assessed using the area under the receiver operating characteristic curve (AUC).

All statistical tests were two-sided, and a P value < 0.05 was considered statistically significant. Statistical analyses were conducted primarily with SPSS version 25.0 (IBM Corp., Armonk, NY, USA). The forest plot was generated using R software (version 3.6.3).

## Results

### Participants characteristics

A total of 2,669 patients with culture-confirmed bacterial infections were included in this retrospective cohort study. Baseline clinical characteristics are summarized in **Table 1**. In the overall cohort, the mean age was 61.16 ± 18.79 years, and 67% of the patients were male. The most prevalent comorbidity was gastrointestinal diseases (42.8%), followed by tumors (26.0%), trauma (22.4%), and hypertension (19.2%). Cardiovascular disease and cerebrovascular disease were present in 20.0% and 14.2% of patients, respectively. Regarding clinical status at admission, fever and abdominal pain were reported as primary symptoms in 4.5% and 7.0% of patients. Severe manifestations such as disorders of consciousness and dyspnea were observed in 2.3% and 5.6% of the cohort, respectively. The majority of patients (93.8%) did not present with acute critical syndromes upon admission; these individuals were admitted for medically necessary reasons including scheduled surgeries, oncologic care, acute-on-chronic disease exacerbations, and complex diagnostic evaluations that required inpatient management but did not constitute immediately life-threatening conditions. The most common admission departments were General Surgery (20.0%), Intensive Care Unit (18.0%), and Orthopedics (17.5%). Prolonged hospitalization (>14 days) was observed in 92.2% of patients. This high proportion reflects the selected nature of the cohort, which included only emergency-admitted patients with culture-confirmed hospital-acquired infection (≥48 hours after admission), as well as the complex case-mix of a tertiary referral hospital.

**Table 1:** Clinical characteristics of the cohort.

Characteristic	Total (n=2669)	Pre-pandemic (n=1886)	Cat-B (A) (n=594)	Cat-B (B) (n=189)	*P*
Demographics					
Age (years, mean ± SD)	61.16 ± 18.79	59.17 ± 18.93	65.46 ± 17.68	67.53 ± 17.06	0.000
Male, n (%)	1,794 (67%)	1,268 (67.2%)	390 (65.7%)	136 (72.0%)	0.275
Comorbidities (n, %)					
Hypertension	512 (19.2%)	312 (16.5%)	163 (27.4%)	37 (19.6%)	<0.001
Diabetes	367 (13.8%)	202 (10.7%)	123 (20.7%)	42 (22.2%)	<0.001
Renal insufficiency	366 (13.7%)	238 (12.6%)	106 (17.8%)	22 (11.6%)	0.004
CeVD	380 (14.2%)	230 (12.2%)	123 (20.7%)	27 (14.3%)	<0.001
CVD	535 (20.0%)	302 (16.0%)	184 (31.0%)	49 (25.9%)	<0.001
GI Diseases	1,141 (42.8%)	770 (40.8%)	275 (46.3%)	96 (50.8%)	0.004
Trauma	599 (22.4%)	463 (24.5%)	101 (17.0%)	35 (18.5%)	<0.001
Tumor	695 (26.0%)	473 (25.1%)	173 (29.1%)	49 (25.9%)	0.147
Clinical Status at Admission, n (%)					
Primary Symptoms					
Fever	121 (4.5%)	70 (3.7%)	43 (7.2%)	8 (4.2%)	0.001
Abdominal Pain	188 (7.0%)	145 (7.7%)	37 (6.2%)	6 (3.2%)	0.047
Severe Symptoms					
Disorders of Consciousness	61 (2.3%)	42 (2.2%)	14 (2.4%)	5 (2.6%)	0.927
Dyspnea	149 (5.6%)	83 (4.4%)	56 (9.4%)	10 (5.3%)	<0.001
Acute Critical Syndromes					<0.001
None (no sepsis or shock)	2,504 (93.8%)	1,784 (94.6%)	550 (92.6%)	170 (89.9%)	
Sepsis without Shock	57 (2.1%)	21 (1.1%)	24 (4.0%)	12 (6.3%)	
Septic Shock	16 (0.6%)	8 (0.4%)	8 (1.3%)	0 (0.0%)	
Non-septic Shock	92 (3.4%)	73 (3.9%)	12 (2.0%)	7 (3.7%)	
Admission Department, (n, %)					
General Surgery	528 (19.8%)	348 (18.5%)	132 (22.2%)	48 (25.4%)	0.018
Intensive Care Unit	480 (18.0%)	305 (16.2%)	138 (23.2%)	37 (19.6%)	<0.001
Orthopedics	468 (17.5%)	370 (19.6%)	76 (12.8%)	22 (11.6%)	<0.001
Emergency Department	254 (9.5%)	198 (10.5%)	21 (3.5%)	35 (18.5%)	<0.001
Urology	105 (3.9%)	78 (4.1%)	21 (3.5%)	6 (3.2%)	0.690
Hepatobiliary Surgery	104 (3.9%)	94 (5.0%)	9 (1.5%)	1 (0.5%)	<0.001
Prolonged hospitalization (>14 days), n (%)	2460 (92.2%)	1,727 (91.6%)	562 (94.6%)	171 (90.5%)	0.037

CeVD, Cerebrovascular Disease; CVD, Cardiovascular Disease; GI Diseases, Gastrointestinal Diseases.

Notable differences were observed across the three pandemic phases. Patients in the Cat-B(A) and Cat-B(B) phases were older and had higher proportions of comorbidities such as hypertension, diabetes, and cardiovascular disease compared to the pre-pandemic phase (all *P* < 0.05). The proportion of patients admitted to the ICU was highest during the Cat-B(A) phase (23.2%), while Emergency Department admissions peaked in the Cat-B(B) phase (18.5%). These differences highlight the evolving patient profile across the study period.

### Shifts in infection sites and pathogen spectrum

**Figure 1:**
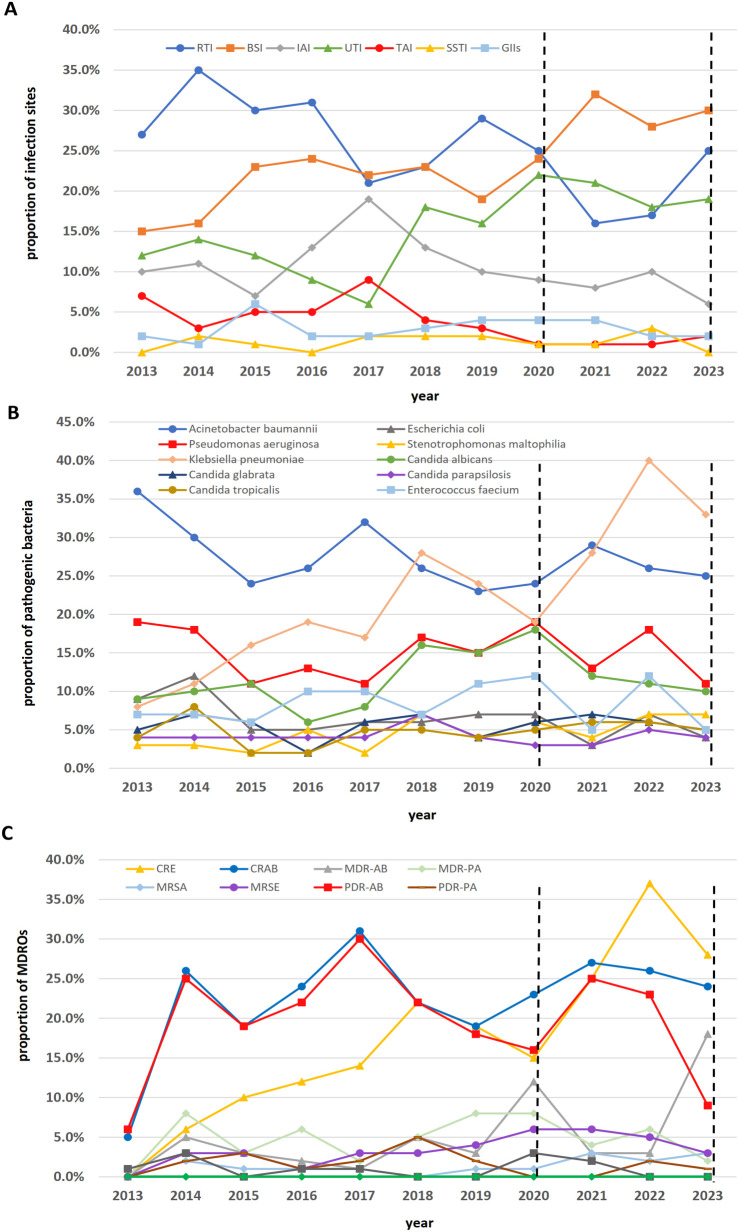
Epidemiological dynamics of healthcare-associated infections across the study period (2013–2023). **(A)** Distribution of infection sites. Line graph showing the annual percentage distribution of cases across seven infection sites: respiratory tract (RTI), bloodstream (BSI), urinary tract (UTI), intra-abdominal (IAI), skin and soft tissue (SSTI), gastrointestinal (GII), and traumatic (TI) infections. **(B)** Prevalence of pathogenic species. Line graph displaying the relative annual abundance (%) of the top isolated bacterial and fungal species. **(C)** Trends in multidrug-resistant organisms. Line graph presenting the annual detection rates (%) of key multidrug-resistant organisms.

The distribution of infection sites exhibited distinct dynamic patterns across the three pandemic phases. In the pre-pandemic period (2013-2019), respiratory tract infections (RTI) were predominant but fluctuated substantially, peaking at 35% in 2014 and declining to 21-23% in 2017-2018. Bloodstream infections (BSI) showed a clear upward trend, rising from 15% in 2013 to a pre-pandemic peak of 24% in 2016, and remained near this level thereafter. Urinary tract infections (UTI) and intra-abdominal infections (IAI) also varied, while skin and soft tissue infections (SSTI) and gastrointestinal infections (GII) remained at consistently low levels (<4%). During the strict containment phase (Cat-B(A), 2020-2022), a marked shift occurred. RTI rates declined sharply to a nadir of 16% in 2021, contrasting with a simultaneous and pronounced surge in BSI, which peaked at 32% in 2021, the highest proportion observed in the entire study period. UTI remained elevated (18-22%), whereas traumatic infections (TAI) nearly vanished (<2%). In the post-adjustment phase (Cat-B(B), 2023), a partial reversion was observed. RTI rebounded to 25%, approximating pre-pandemic and early-pandemic levels. However, BSI remained persistently high at 30%, and UTI stayed elevated at 19%. Proportions of other infection sites remained low and stable (**Figure 1A**).

The ranking of the top ten pathogens revealed Gram-negative bacterial dominance. *Acinetobacter baumannii* continuously declined from a peak of 36% in 2013 to 25% in 2023. In contrast, *Klebsiella pneumoniae* became more common during Cat-B(A), peaking at 40% in 2022. *Pseudomonas aeruginosa* detection remained stable (1-19%). Proportions of *Enterococcus faecium* varied between 5% and 12% across the study years. The combined proportion of remaining pathogens was <15% with no significant temporal trend (*P* > 0.05) (**Figure 1B**).

**Figure 1C** illustrates the shifting epidemiology of key multidrug-resistant organisms across the study period. CRE showed an increase, particularly during the Cat-B(A) phase. Its detection rate increased to 37% in 2022, the highest observed, before slightly declining to 28% in 2023.In contrast, CRAB demonstrated a different pattern. It was the predominant resistant pathogen in the pre-pandemic years, peaking at 31% in 2017. During the pandemic phases, its prevalence showed fluctuations but remained at substantial levels, ranging between 22% and 27%, and was 24% in 2023.Notably, PDR-AB prevalence declined from a pre-pandemic peak of 25-30% to 9% in 2023. Conversely, multidrug-resistant *A. baumannii* (MDR-AB) showed a pronounced increase in 2023, reaching 18%, which contrasted with its generally low prevalence (<5%) in preceding years. Others resistant phenotypes, including MDR-PA, MRSA, and VRE, were detected at consistently low rates (generally below 5-8%) throughout the period, without a clear temporal trend. VRSA was not detected in any year. In addition, the distribution of the number of MDRO species per patient is shown in **Supplementary Table S1**. Before the pandemic, the majority of patients (63.1%) were not infected or colonized by any MDRO. This proportion dropped significantly to 43.6% in the Cat-B (A) sub-phase and then rebounded to 51.3% in the Cat-B (B) sub-phase. Concurrently, the proportion of patients carrying a single MDRO species increased significantly from 14.5% pre-pandemic to 27.6% in Cat-B (A). Similarly, the proportion carrying three or more MDRO species increased from 4.5% to 8.4%. Notably, the prevalence of both single-species and poly-species (≥3) MDRO carriage peaked during the Cat-B (A) sub-phase.

### MDRO site distribution shifts during COVID-19 policy changes

Pre-pandemic, CRE detection was significantly higher in IAI (19.4%) compared to non-IAI cases (10.9%, *P<*0.001). In UTI, the prevalence of CRAB and PDR-AB was significantly lower, while VRE was more common (all *P* < 0.001). During Cat-B(A), site-specific differences were observed. In RTI, the prevalence of both CRAB (39.0% vs. 23.1%, *P* = 0.001) and PDR-AB (37.1% vs. 19.2%, *P* < 0.001) increased markedly. Conversely, in UTI, CRE became more prevalent (35.5% vs. 24.5%, P = 0.014), while CRAB and PDR-AB were less common (*P* < 0.001). Additionally, CRE was more frequently detected with IAI (49.1% vs. 24.5%, *P* < 0.001), and MRSA with BSI (4.7% vs. 1.2%, *P* = 0.009). In the Cat-B(B) phase, the prevalence of CRAB and PDR-AB in BSI was significantly lower than in non-BSI cases (*P* < 0.05). The previously strong associations between RTI and CRAB/PDR-AB were no longer statistically significant (**Table 2**).

**Table 2:** Variations in MDRO prevalence at different infection sites throughout pandemic periods [n (%)].

Infection sites	CRAB	CRE	MRSA	PDR-PA	VRE	PDR-AB	Others
Pre-pandemic							
RTI							
Yes	106 (19.9)	43 (8.1)	4 (0.7)	8 (1.5)	0 (0.00)	102 (19.1)	51 (9.6)
No	285 (21.1)	181 (13.4)	15 (1.1)	31 (2.3)	14 (1.0)	278 (20.6)	124 (9.2)
*P*	0.553	0.001	0.480	0.275	0.018	0.476	0.798
BI							
Yes	76 (19.9)	56 (14.7)	7 (1.8)	5 (1.3)	4 (1.0)	73 (19.2)	42 (11.0)
No	315 (20.9)	168 (11.2)	12 (0.8)	34 (2.3)	10 (0.7)	307 (20.4)	133 (8.8)
*P*	0.673	0.057	0.069	0.246	0.434	0.59	0.189
UTI							
Yes	17 (7.2)	31 (13.1)	0 (0.00)	7 (3.0)	7 (3.0)	16 (6.8)	9 (3.8)
No	374 (22.7)	193 (11.7)	19 (1.2)	32 (1.9)	7 (0.4)	364 (22.1)	166 (10.1)
*P*	<0.001	0.523	0.098	0.300	<0.001	<0.001	0.002
IAI							
Yes	45 (20.8)	42 (19.4)	1 (0.5)	5 (2.3)	0 (0.00)	45 (20.8)	22 (10.2)
No	346 (20.7)	18 (10.9)	18 (1.1)	34 (2.0)	14 (0.8)	335 (20.1)	153 (9.2)
*P*	0.969	<0.001	0.394	0.786	0.177	0.790	0.626
TI							
Yes	24 (24.7)	12 (12.4)	2 (2.1)	3 (3.1)	1 (1.0)	23 (23.7)	8 (8.2)
No	367 (20.5)	212 (11.9)	17 (1.0)	36 (2.0)	13 (0.7)	357 (20.0)	167 (9.3)
*P*	0.317	0.877	0.286	0.466	0.734	0.369	0.719
Cat-B (A)							
RTI							
Yes	41 (39.0)	16 (15.2)	1 (1.0)	0 (0.00)	0 (0.00)	39 (37.1)	20 (19.0)
No	113 (23.1)	143 (29.2)	12 (2.5)	4 (0.8)	11 (2.2)	94 (19.2)	74 (15.1)
*P*	0.001	0.003	0.340	0.352	0.121	<0.001	0.319
BI							
Yes	23 (13.4)	38 (22.1)	8 (4.7)	0 (0.00)	0 (0.00)	18 (10.5)	34 (19.8)
No	131 (31.0)	121 (28.7)	5 (1.2)	4 (0.9)	11 (2.6)	115 (27.3)	60 (14.2)
*P*	<0.001	0.100	0.009	0.200	0.033	<0.001	0.093
UTI							
Yes	9 (7.3)	44 (35.5)	0 (0.00)	0 (0.00)	6 (4.8)	8 (6.5)	7 (5.6)
No	145 (30.9)	115 (24.5)	13 (2.8)	4 (0.9)	5 (1.1)	125 (26.6)	87 (18.5)
*P*	<0.001	0.014	0.061	0.303	0.06	<0.001	<0.001
IAI							
Yes	18 (32.7)	27 (49.1)	0 (0.00)	3 (5.5)	2 (3.6)	16 (29.1)	85 (15.8)
No	136 (25.2)	132 (24.5)	13 (2.4)	1 (0.2)	9 (1.7)	117 (21.7)	9 (16.4)
*P*	0.227	<0.001	0.244	<0.001	0.303	0.211	0.909
TI							
Yes	1 (20.0)	2 (40. 0)	0 (0.00)	0 (0.00)	0 (0.00)	1 (20. 0)	93 (15.8)
No	153 (26.0)	157 (26.7)	13 (2.2)	4 (0.7)	11 (1.9)	132 (22.4)	1 (20.0)
*P*	0.761	0.502	0.737	0.853	0.758	0.898	0.797
Cat-B (B)							
RTI							
Yes	15 (30.0)	13 (26. 0)	1 (2.0)	0 (0.00)	0 (0.00)	4 (8.0)	15 (30.0)
No	29 (20.9)	38 (27.3)	4 (2.9)	1 (0.7)	0 (0.00)	11 (7.9)	26 (18.7)
*P*	0.190	0.855	0.740	0.548	0 (0.00)	0.985	0.097
BI							
Yes	7 (12.3)	9 (15.8)	0 (0.00)	0 (0.00)	0 (0.00)	1 (1.8)	11 (19.3)
No	37 (28.0)	42 (31.8)	5 (3.8)	1 (0.8)	0 (0.00)	14 (10.6)	30 (22.7)
*P*	0.019	0.023	0.136	0.510	0 (0.00)	0.039	0.600
UTI							
Yes	1 (2.8)	12 (33.3)	0 (0.00)	0 (0.00)	0 (0.00)	1 (2.8)	0 (0.00)
No	42 (28.1)	39 (25.5)	5 (3.3)	1 (0.7)	0 (0.00)	14 (9.2)	41 (26.8)
*P*	0.001	0.340	0.272	0.627	0 (0.00)	0.203	<0.001
IAI							
Yes	2 (18.2)	4 (36.4)	1 (9.1)	0 (0.00)	0 (0.00)	2 (18.2)	40 (22.5)
No	42 (23.6)	47 (26.4)	4 (2.2)	1 (0.6)	0 (0.00)	13 (7.3)	1 (9.1)
*P*	0.680	0.470	0.170	0.803	0 (0.00)	0.195	0.296
TI							
Yes	2 (66.7)	0 (0.00)	0 (0.00)	0 (0.00)	0 (0.00)	1 (33.3)	40 (21.5)
No	42 (22.6)	51 (27.4)	5 (2.7)	1 (0.5)	0 (0.00)	14 (7.5)	1 (33.3)
*P*	0.073	0.289	0.773	0.900	0 (0.00)	0.101	0.622

RTI, Respiratory Tract Infection; BI, Bloodstream Infection; UTI, Urinary Tract Infection; IAI, Intra-abdominal infection; TI, Traumatic Infection.

### Risk factors for MDRO infection

**Figure 2:**
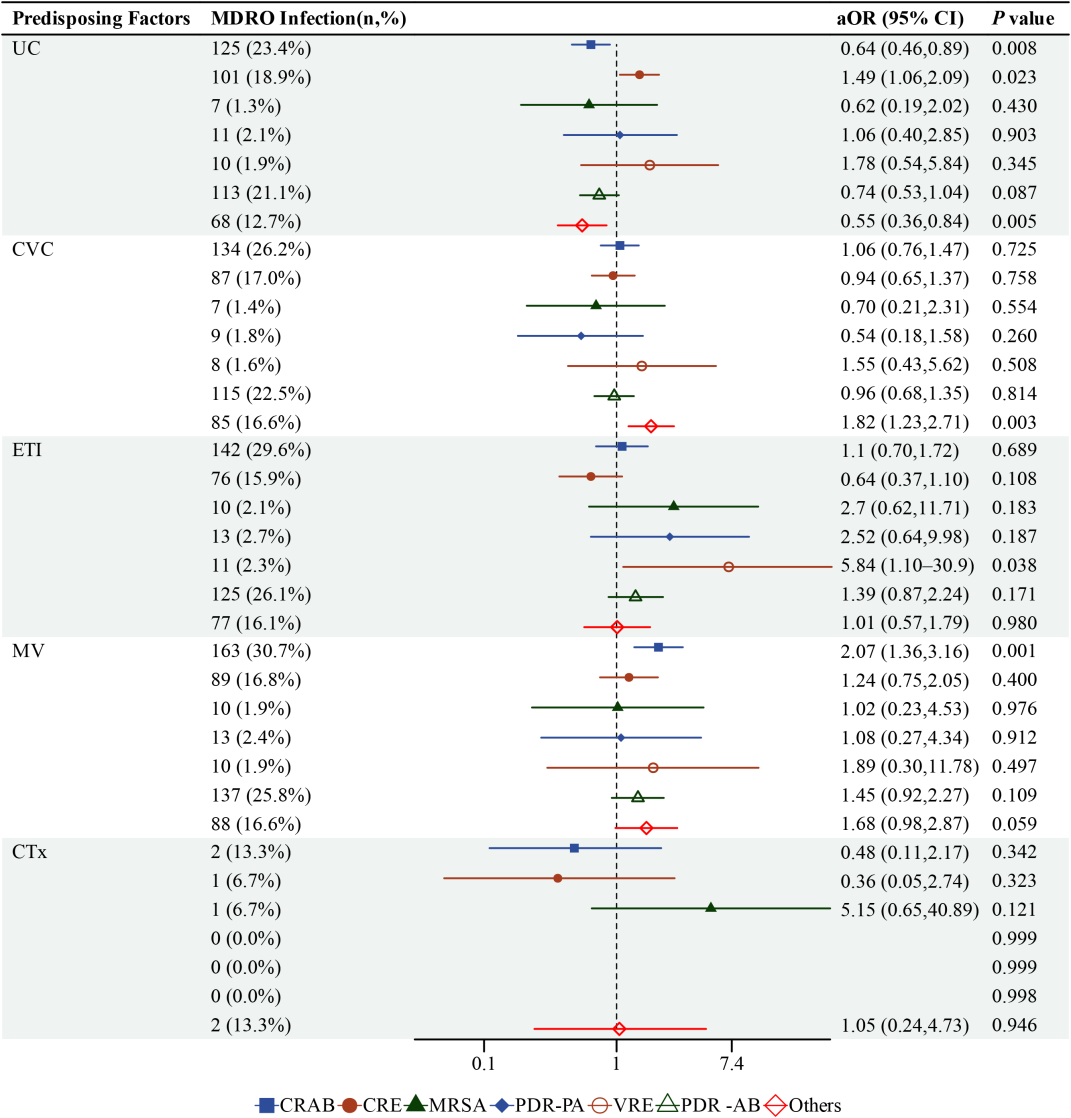
Forest plot of multivariate analysis for risk factors associated with MDRO infections. Adjusted odds ratios (aORs) and 95% confidence intervals (CIs) are displayed for each invasive procedure across specific MDRO types. All models were adjusted for age, sex, comorbidities, clinical status at admission, admission department, and prolonged hospitalization including UC, urinary catheter; CVC, central venous catheter; ETI, endotracheal intubation; MV, mechanical ventilation; CTx, chemotherapy.

The detailed results of the multivariate logistic regression analysis are presented in **Supplementary Table S2** and visualized in **Figure 2**. Mechanical ventilation (MV) was associated with significantly increased odds of CRAB infection (aOR=2.07, 95%CI:1.36-3.16). Urinary catheterization (UC) demonstrated divergent effects, showing significant negative associations with CRAB (aOR=0.64, 95% CI: 0.46-0.89) and infections by “Other” pathogens (aOR=0.55, 95% CI: 0.36-0.84), while concurrently presenting as a risk factor for CRE infection (aOR=1.49, 95%CI: 1.06-2.09). Central venous catheterization (CVC) was significantly associated with increased odds of “Other” MDRO infections (aOR=1.82, 95% CI:1.23-2.71). Notably, endotracheal intubation (ETI) was associated with significantly increased odds of VRE infection (aOR=5.84, 95% CI: 1.10-30.9). For MRSA, PDR-PA, and PDR-AB infections, none of the examined invasive procedures showed statistically significant associations. Chemotherapy (CTx) was not significantly associated with any MDRO infection category. Model calibration was acceptable for most regression models, whereas discrimination was modest; detailed model performance measures are provided in **Supplementary Table S3**.

### Epidemiological characteristics and risk factors for CRAB and CRE infections in hospitalized patients

**Figure 3:**
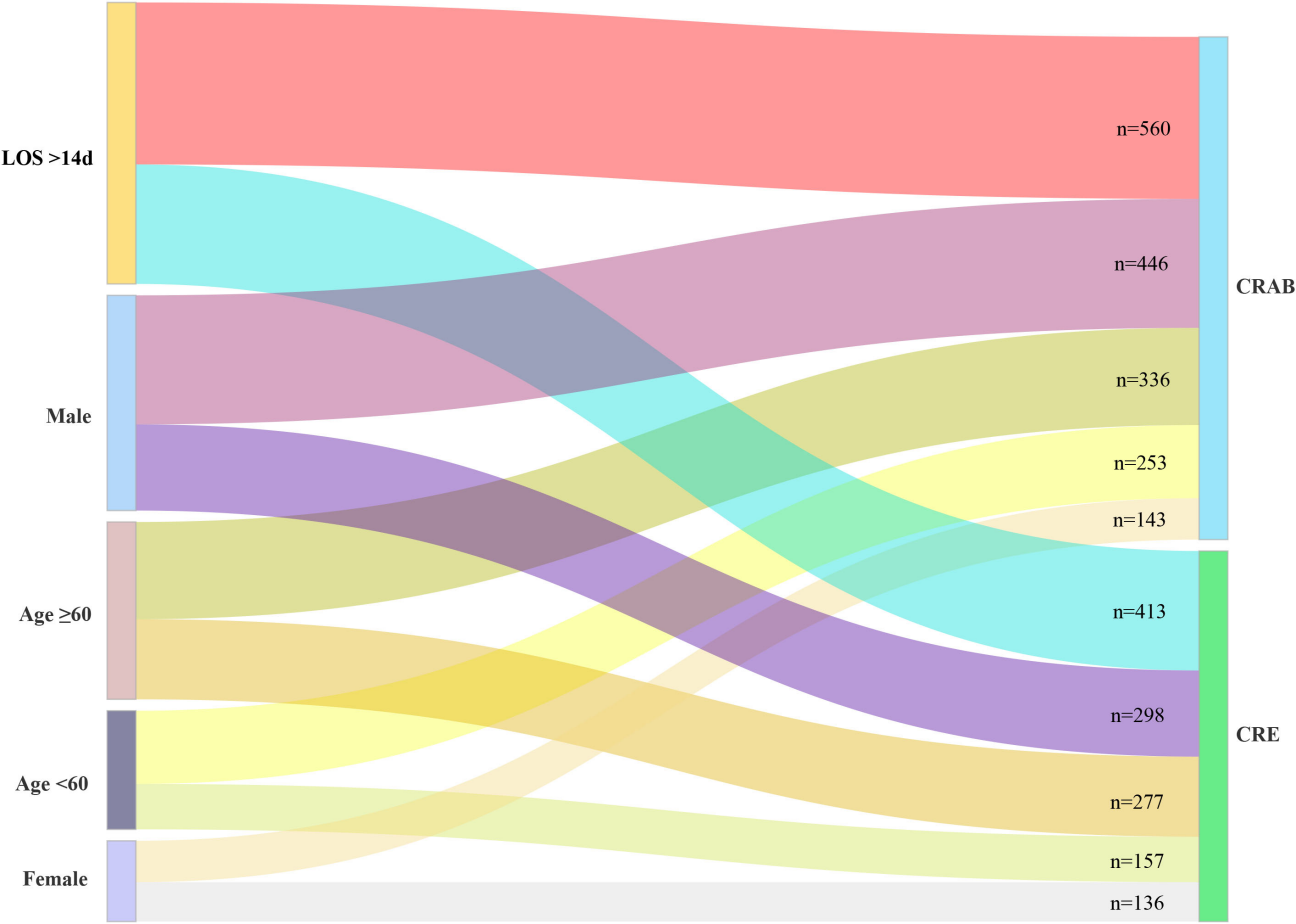
Sankey diagram of patient flow from key risk groups to CRAB or CRE infection outcomes. The diagram visualizes the quantitative pathways from major demographic (sex, age) and clinical (length of hospital stay) risk groups (left) to the two predominant multidrug-resistant infection outcomes, CRAB and CRE (right). Node labels indicate the total number of patients (n) in each category. The width of each flow link is proportional to the number of patients.

As shown in **Supplementary Table S4**, CRAB and CRE remained the two predominant multidrug-resistant organism (MDRO) types across the study period, with CRE showing a marked increase during the Cat-B(A) and Cat-B(B) phases. Specifically, CRE detection increased from 11.9% in the pre-pandemic phase to 26.8% in Cat-B(A) and 27.0% in Cat-B(B), whereas CRAB remained relatively stable at substantial levels across phases. The distribution of these two major MDROs across demographic and clinical subgroups is summarized in **Supplementary Table S5**. Male patients had a higher proportion of CRAB infection than female patients (24.9% vs. 16.3%, P < 0.001), whereas patients aged ≥60 years had a higher proportion of CRE infection than younger patients (17.6% vs. 14.3%, P = 0.024). In addition, patients with prolonged hospitalization (>14 days) had higher proportions of both CRAB and CRE infection than those with shorter stays. Based on these subgroup distributions, **Figure 3** visually summarizes the flow patterns from major demographic and clinical subgroups to the two predominant MDRO outcomes, CRAB and CRE.

## Discussion

All 2,669 patients in this study were recruited from the emergency department (ED) of a single large tertiary teaching hospital. As a regional medical center, our ED manages a high volume of cases with complex conditions, admitting a substantial number of patients with acute, undiagnosed infections requiring urgent hospitalization. This enables our cohort to reasonably reflect the true epidemiological burden and risk profile of acute bacterial infections, particularly MDROs, among patients first presenting to the ED in our region. This study showed substantial changes in infection-site distribution, pathogen spectrum, and procedure-associated risk patterns across the COVID-19 policy phases. Firstly, there was a shift in the spectrum of infection sites, with the proportion of bloodstream infections increasing significantly and persistently. Secondly, the ecological niche of pathogens changed, with CRE replacing CRAB as the dominant pathogen. Finally, the precise identification was made of the highly specific clinical risk pathways that drive different types of antibiotic-resistant infections. This suggests that in the post-pandemic era, infection control may need to upgrade from “universal prevention” to “targeted intervention” based on precise matching of pathogens and risk pathways.

Our study demonstrated significant shifts in infection site prevalence during the COVID-19 pandemic, characterized by a rebound in RTI and a concerning rise in BSI. In the pre-pandemic period, RTI peaked at 35.0% in 2014. During strict containment, their rates initially declined but subsequently rebounded to pre-pandemic levels upon policy relaxation, a trend consistent with epidemiological studies reporting a rebound of respiratory pathogens following the relaxation of COVID-19 non-pharmaceutical interventions (Zhao et al., 2024). Concurrently, we observed a concerning rise in the proportion of BSI from 24% to 32% during the strict containment phase. This finding contrasts with a study in hematological malignancy patients under enhanced protection (Cai et al., 2023) but aligns with reports from critically ill COVID-19 cohorts (Bonazzetti et al., 2023), suggesting that the increased baseline vulnerability of patients admitted during the pandemic was a contributing factor.

A pivotal epidemiological shift was the rise of *K.pneumoniae* and CRE, which supplanted CRAB as the dominant pathogens. *K.pneumoniae* infection rates peaked at 40.0% in 2022, mirroring its increased isolation in other settings during the pandemic (Hao et al., 2023) and reflecting a global trend of heightened MDRO transmission in COVID-19 epicenters (Kolbe-Busch et al., 2025). The divergent trajectories of CRE and CRAB are particularly instructive. While CRAB was the pre-pandemic dominant MDRO (Li et al., 2021), its dominance waned during strict containment, whereas CRE accelerated, peaking at 37% in 2022. This divergence likely stems from their distinct transmission mechanisms. Strengthened infection control, particularly stringent environmental disinfection, likely effectively limited the environmental transmission typical of CRAB. In contrast, CRE colonization is frequently present at admission and can lead to subsequent hospital-acquired infections. This indicates that strain importation, a process less impacted by intra-hospital NPIs (Li et al., 2021), is a key driver of CRE spread, which explains the rise in incidence despite enhanced precautions. The integration of descriptive epidemiological trends with multivariable regression findings provides a more nuanced understanding of these dynamics. The observed shift from CRAB to CRE during the strict containment phase (**Figure 1C**) aligns with the procedural risk factors identified in our regression analysis: urinary catheterization emerged as a significant risk factor for CRE (aOR=1.49, 95% CI: 1.06–2.09), whereas mechanical ventilation was specifically associated with CRAB (aOR=2.07, 95% CI: 1.36–3.16). These findings suggest that the changing prevalence of these pathogens may be partly attributable to shifts in the utilization of specific invasive procedures across phases, highlighting the need for pathogen-tailored prevention strategies.

Most notably, our study revealed procedure-specific risk patterns that directly inform bedside prevention and guide empiric therapy. The strong association between mechanical ventilation and CRAB infection is supported by a UK research, indicating that specific ventilator settings can influence physiological responses and infection risk, positioning optimized ventilation as a critical targeted intervention (Mumtaz et al., 2023). This is especially relevant in complex clinical scenarios such as COVID-19, where co-infection or superinfection has been associated with worse outcomes, including higher mortality and greater use of mechanical ventilation (Musuuza et al., 2021). Furthermore, the elevated CRE risk associated with urinary catheterization is consistent with studies showing that indwelling urinary catheters increase the risk of subsequent CRE bacteremia and other healthcare-associated resistant Enterobacterales infections (Howard-Anderson et al., 2021). Of particular clinical relevance, endotracheal intubation was associated with a markedly increased risk of VRE infections (aOR = 5.84, 95% CI: 1.10–30.9, *P* = 0.038). This finding may be biologically plausible, as patients requiring intubation often experience severe illness, prolonged ICU exposure, and broad-spectrum antibiotic use, all of which may promote intestinal colonization and subsequent infection with vancomycin-resistant enterococci. However, this estimate should be interpreted cautiously given the relatively small number of VRE cases. Additionally, patients requiring intubation often receive broad-spectrum antibiotics, including anti-anaerobic agents, which can disrupt the gut microbiota and promote intestinal domination by vancomycin-resistant enterococci (Ubeda et al., 2010; King et al., 2025). Preclinical and clinical studies have demonstrated that antibiotic treatment enables VRE to displace the normal microbiota and establish intestinal colonization, which can precede bloodstream infection (Ubeda et al., 2010). Furthermore, antibiotics create a nutrient-rich environment in the gut that favors VRE growth by increasing available carbon and nitrogen sources while reducing inhibitory microbial metabolites (King et al., 2025).

In addition, our analysis identified specific risk factors for precision infection control. Consistent with literature, our study confirmed that susceptibility to MDROs is demographically patterned, with male patients being more vulnerable to CRAB and PDR-AB (Kodde et al., 2025), and elderly patients (≥60 years) to CRE and MRSA (Avci et al., 2012; Denkinger et al., 2013). Prolonged hospitalization also emerged as a critical risk factor. Patients hospitalized for more than 14 days had substantially higher rates of CRAB, CRE, and PDR-AB infections, with a clear dose-response relationship between length of stay and infection risk. Accordingly, the frequency of prolonged hospitalization in this study should not be interpreted as representative of the general inpatient population, but rather of a clinically complex subgroup at high risk for nosocomial infection. Patients with prolonged ICU stay are at high risk of MDRO infection, which may be aggravated by factors such as nutritional compromise and invasive procedures (Blanco et al., 2018; Rong et al., 2022). The use of mechanical ventilation for more than 14 days is associated with higher rates of ICU-acquired infections, which are often difficult to treat due to antibiotic resistance (Lin et al., 2015). The presence of MDR bacteria in ICU settings, particularly during the COVID-19 pandemic, has also been noted to increase, highlighting the need for stringent infection control measures (Bogossian et al., 2020). Beyond host and iatrogenic risks, antibiotic management practices and antimicrobial stewardship may also influence the development and spread of resistant infections (Kengkla et al., 2022; Ya et al., 2023).

Several limitations should be considered when interpreting these results. First, the single-center design may limit generalizability to other healthcare settings, and the observational nature of our study precludes definitive causal inferences; residual confounding may exist despite multivariable adjustment. Second, data on antibiotic exposure prior to infection, a known driver of MDRO acquisition, were not available for analysis. Third, several pandemic-related factors could have influenced the observed findings: changes in sampling practices may have affected detection rates; shifts in ward composition and patient mix may have altered transmission dynamics; and changes in patient case mix, emergency department admission thresholds, and ICU occupancy rates may have confounded the observed differences across phases. These potential biases suggest that our findings should be interpreted as associations rather than causal relationships. Despite these limitations, this study has notable strengths. The 10-year study period, spanning the pre-pandemic, strict containment, and post-adjustment phases, provides a unique longitudinal perspective on MDRO dynamics during a major healthcare disruption. The large sample size (2,669 patients) and comprehensive clinical data allowed for robust multivariable modeling and identification of pathogen-specific risk factors. Furthermore, the focus on patients admitted through the emergency department ensures that our cohort reflects real-world clinical practice and the acute infection burden in our region.

## Conclusion

Our longitudinal data reveal a consequential shift in hospital-acquired MDRO epidemiology during the pandemic period. The most prominent change was the rise of CRE to become the dominant resistant pathogen, a trend especially evident among older patients and those requiring urinary catheters. We further established that prolonged hospitalization acts as a key risk amplifier. Within this context, specific invasive procedures emerged as decisive, pathogen-specific drivers: mechanical ventilation for CRAB and urinary catheterization for CRE. These insights create a practical imperative for precision in infection control. Rather than applying broad interventions uniformly, our findings support strategies tailored to specific pathogen-procedure-patient combinations. In practice, this means prioritizing rigorous ventilator bundles and environmental cleaning for ventilated patients at high risk for CRAB, while simultaneously strengthening catheter care and considering early screening for CRE in catheterized elderly patients. Validating the efficacy of these targeted intervention bundles in prospective studies is the essential next step to mitigate the burden of antimicrobial resistance.

## Future directions

Building on the findings of this single-center study, several key directions for future research emerge. First, continued surveillance is warranted to evaluate whether the post-pandemic shift in pathogen dominance, particularly the rise of CRE, is sustained or transient, and how future public health emergencies might influence resistance patterns. Second, multicenter studies across diverse geographic regions and hospital types in China are essential to validate the generalizability of our risk factor models and to capture a more comprehensive picture of the national MDRO landscape.

## Data Availability

The raw data supporting the conclusions of this article will be made available by the authors, without undue reservation.
