# Supplementary material for: Epidemiology and risk factors of multidrug-resistant organisms in a Chinese tertiary hospital: a 10-year retrospective cohort study across the COVID-19 policy phases, 2013–2023
Source: Front Cell Infect Microbiol. 2026 Apr 7;16:1786780. doi: 10.3389/fcimb.2026.1786780 (PMC13095828; doi:10.3389/fcimb.2026.1786780)
Supplement: Supplementary file 1 [file Table1.docx]

**Table S1** Number of MDRO species [n (%)]

| **Count** | **Pre-pandemic（n=1886）** | **Cat-B (A)**  **（n=594）** | **Cat-B (B)（n=189）** | **χ²** | ***P*** |
| --- | --- | --- | --- | --- | --- |
| **0** | 1191（63.1） | 259（43.6） | 97（51.3） | 96.17 | < 0.001 |
| **1** | 274（14.5） | 164（27.6） | 46（24.3） |  |  |
| **2** | 311（16.5） | 114（19.2） | 29（15.3） |  |  |
| **3** | 85（4.5） | 50（8.4） | 13（6.9） |  |  |
| **≥4** | 25（1.4） | 7（1.2） | 4（2.2） |  |  |

**Table S2** Multivariate analysis of predisposing factors and MDRO infections [n (%)]

| **Predisposing Factors** | **CRAB** | **CRE** | **MRSA** | **PDR-PA** | **VRE** | **PDR -AB** | **Others** |
| --- | --- | --- | --- | --- | --- | --- | --- |
| **UC** | 125（23.4） | 101（18.9） | 7（1.3） | 11（2.1） | 10（1.9） | 113（21.1） | 68（12.7） |
| *P* | 0.008 | 0.023 | 0.430 | 0.903 | 0.345 | 0.087 | 0.005 |
| OR | 0.64（0.46-0.89） | 1.49（1.06-2.09） | 0.62 (0.19-2.02) | 1.06 (0.40-2.85) | 1.78（0.54-5.84） | 0.74 (0.53-1.04) | 0.55 (0.36-0.84) |
| **CVC** | 134（26.2） | 87（17.0） | 7（1.4） | 9（1.8） | 8（1.6） | 115（22.5） | 85（16.6） |
| *P* | 0.725 | 0.758 | 0.554 | 0.260 | 0.508 | 0.814 | 0.003 |
| OR | 1.06（0.76-1.47） | 0.94（0.65-1.37） | 0.70 (0.21-2.31) | 0.54 (0.18-1.58) | 1.55（0.43-5.62） | 0.96 (0.68-1.35) | 1.82 (1.23-2.71) |
| **ETI** | 142（29.6） | 76（15.9） | 10（2.1） | 13（2.7） | 11（2.3） | 125（26.1） | 77（16.1） |
| *P* | 0.689 | 0.108 | 0.183 | 0.187 | 0.038 | 0.171 | 0.980 |
| OR | 1.10（0.70-1.72） | 0.64（0.37-1.10） | 2.70 (0.62-11.71) | 2.52 (0.64-9.98) | 5.84 (1.10-30.9) | 1.39 (0.87-2.24) | 1.01 (0.57-1.79) |
| **MV** | 163（30.7） | 89（16.8） | 10（1.9） | 13（2.4） | 10（1.9） | 137（25.8） | 88（16.6） |
| *P* | 0.001 | 0.400 | 0.976 | 0.912 | 0.497 | 0.109 | 0.059 |
| OR | 2.07（1.36-3.16） | 1.24 (0.75-2.05) | 1.02 (0.23-4.53) | 1.08 (0.27-4.34) | 1.89 (0.30-11.78） | 1.45 (0.92-2.27) | 1.68 (0.98-2.87) |
| **CTx** | 2（13.3） | 1（6.7） | 1（6.7） | 0（0.0） | 0（0.0） | 0（0.0） | 2（13.3） |
| *P* | 0.342 | 0.323 | 0.121 | 0.999 | 0.999 | 0.998 | 0.946 |
| OR | 0.48（0.11-2.17） | 0.36（0.05-2.74） | 5.15（0.65-40.89） | 0（0.00） | 0（0.00） | 0（0.00） | 1.05（0.24-4.73） |

Urinary Catheterization (UC), Central Venous Catheterization (CVC), Endotracheal Intubation (ETI), Mechanical Ventilation (MV), Chemotherapy for Tumors (CTx). *P*-values are derived from multivariate logistic regression. Dashes (-) denote non-calculable data; all models were confounder-adjusted. All models were adjusted for age, sex, comorbidities, clinical status at admission, admission department, and prolonged hospitalization.

**Table S3** Performance of multivariable logistic regression models for each MDRO outcome.

| **MDRO** | **Hosmer-Lemeshow χ²** | **df** | ***P*** | **AUC** | **95% CI** |
| --- | --- | --- | --- | --- | --- |
| **VRE** | 0.736 | 2 | 0.692 | 0.67 | 0.56-0.78 |
| **PDR-PA** | 3.528 | 3 | 0.317 | 0.58 | 0.49-0.66 |
| **MRSA** | 2.847 | 3 | 0.416 | 0.57 | 0.47-0.68 |
| **CRAB** | 6.731 | 3 | 0.081 | 0.56 | 0.54-0.59 |
| **Others** | 0.693 | 2 | 0.707 | 0.56 | 0.53-0.60 |
| **PDR-AB** | 12.513 | 3 | 0.006 | 0.56 | 0.54-0.59 |
| **CRE** | 0.421 | 2 | 0.810 | 0.53 | 0.50-0.56 |

**Table S4** Detection of MDRO [n (%)]

| **Species** | **Pre-pandemic** | **Cat-B (A)** | | | **Cat-B (B)** | **χ²** | | ***P*** |  |
| --- | --- | --- | --- | --- | --- | --- | --- | --- | --- |
| MDRO | 695（36.9） | | 335（56.4） | 92（49.2） | | 74.51 | | < 0.001 |  |
| CRAB | 391（20.7） | | 154（25.9） | 44（23.3） | | 7.26 | | 0.027 |  |
| CRE | 224（11.9） | | 159（26.8） | 51（27.0） | | 90.74 | | < 0.001 |  |
| MRSA | 19（1.0） | | 13（2.2） | 5（2.6） | | 6.97 | | 0.031 |  |
| PDR-PA | 39（2.1） | | 4（0.7） | 1（0.5） | | 6.99 | | 0.030 |  |
| VRE | 14（0.7） | | 11（1.9） | 0（0.00） | | 7.92 | | 0.019 |  |
| PDR-AB | 380（20.1） | | 133（22.4） | 15（7.9） | | 19.42 | | < 0.001 |  |
| MDR-AB | 53（2.8） | | 32（5.4） | 34（18.0） | | 94.46 | < 0.001 | | |
| MDR-PA | 89（4.7） | | 32（5.4） | 4（2.1） | | 3.45 | 0.178 | | |
| MRSE | 45（2.4） | | 32（5.4） | 5（2.6） | | 13.79 | 0.001 | | |
| VRSA | 1（0.1） | | 0（0.00） | 0（0.00） | | 0.42 | 0.812 | | |

**Table S5** Comparison of MDRO Infections Across Sex, Age, and Hospital Stay Duration [n (%)]

| **Grouping** | **CRAB** | **CRE** | **MRSA** | **PDR-PA** | **VRE** | **PDR -AB** | **Others** |  |
| --- | --- | --- | --- | --- | --- | --- | --- | --- |
| **Gender** |  |  |  |  |  |  |  |  |
| Male | 446（24.9） | 298（16.6） | 25（1.4） | 31（1.7） | 14（0. 8） | 399（22.2） | 233（13.0） |  |
| Female | 143（16.3） | 136（15.5） | 12（1.4） | 13（1.5） | 11（1.3） | 129（14.7） | 77（8.8） |  |
| *P* | ＜0.001 | 0.483 | 0.963 | 0.644 | 0.230 | ＜0.001 | 0.002 |  |
| **Age** |  |  |  |  |  |  |  |  |
| ＜60 | 253（23.1） | 157（14.3） | 8（0.7） | 18（1.6） | 6（0.5） | 236（21.5） | 126（11.5） |  |
| ≥60 | 336（21.4） | 277（17.6） | 29（1.8） | 26（1.7） | 19（1.2） | 292（18.6） | 184（11.7） |  |
| *P* | 0.291 | 0.024 | 0.015 | 0.983 | 0.081 | 0.058 | 0.873 |  |
| **LOS** | |  |  |  |  |  |  |  |
| ＜7 d | 7（33.3） | 2（9.5） | 0（0.00） | 1（4.8） | 0（0.00） | 5（23.8） | 3（14.3） |  |
| 7-14 d | 22（11.7） | 19（10.1） | 2（1.1） | 0（0.00） | 1（0.5） | 20（10.6） | 10（5.3） |  |
| ＞14 d | 560（22.8） | 413（16.8） | 35（1.4） | 43（1.7） | 24（1.0） | 503（20.4） | 297（12.1） |  |
| *P* | 0.001 | 0.040 | 0.794 | 0.102 | 0.752 | 0.005 | 0.019 |  |

Length of Hospital Stay (LOS)
